# Analysis of an alternative human CD133 promoter reveals the implication of Ras/ERK pathway in tumor stem-like hallmarks

**DOI:** 10.1186/1476-4598-9-39

**Published:** 2010-02-19

**Authors:** Kouichi Tabu, Taichi Kimura, Ken Sasai, Lei Wang, Norihisa Bizen, Hiroshi Nishihara, Tetsuya Taga, Shinya Tanaka

**Affiliations:** 1 Laboratory of Cancer Research, Department of Pathology, Hokkaido University Graduate School of Medicine, Kita-15, Nishi-7, Kita-ku, Sapporo, Japan; 2 Department of Stem Cell Regulation, Medical Research Institute, Tokyo Medical and Dental University, 1-5-45 Yushima, Bunkyo-ku, Tokyo, Japan; 3 Global COE Program "Cell Fate Regulation Research and Education Unit", Kumamoto University, 2-2-1 Honjo, Kumamoto, Japan; 4 Division of Cell Fate Modulation, Institute of Molecular Embryology and Genetics, Kumamoto University, 2-2-1 Honjo, Kumamoto, Japan; 5 Research Group for Human Cell Transformation, KAN Research Institute Inc., Kobe MI R&D Center, 6-7-3 Minatojima-Minamimachi, Chuo-ku, Kobe, Japan; 6 Department of Translational Pathology, Hokkaido University Graduate School of Medicine, Kita-15, Nishi-7, Kita-ku, Sapporo, Japan

## Abstract

**Background:**

An increasing number of studies support the presence of stem-like cells in human malignancies. These cells are primarily responsible for tumor initiation and thus considered as a potential target to eradicate tumors. CD133 has been identified as an important cell surface marker to enrich the stem-like population in various human tumors. To reveal the molecular machinery underlying the stem-like features in tumor cells, we analyzed a promoter of *CD133 *gene using human colon carcinoma Caco-2 and synovial sarcoma Fuji cells, which endogenously express *CD133 *gene.

**Results:**

A reporter analysis revealed that P5 promoter, located far upstream in a human *CD133 *gene locus, exhibits the highest activity among the five putative promoters (P1 to P5). Deletion and mutation analysis identified two ETS binding sites in the P5 region as being essential for its promoter activity. Electrophoretic mobility shift assays demonstrated the specific binding between nuclear factors and the ETS binding sequence. Overexpression of dominant-negative forms of Ets2 and Elk1 resulted in the significant decrease of P5 activity. Furthermore, treatment of Fuji cells with a specific MEK/ERK inhibitor, U0126, also markedly decreased CD133 expression, but there was no significant effect in Caco-2 cells, suggesting cell type-specific regulation of CD133 expression. Instead, the side population, another hallmark of TSLCs, was dramatically diminished in Caco-2 cells by U0126. Finally, Ras-mediated oncogenic transformation in normal human astrocytes conferred the stem-like capability to form neurosphere-like colonies with the increase of *CD133 *mRNA expression.

**Conclusions:**

In conclusion, the Ras/ERK pathway at least in part contributes to the maintenance and the acquisition of stem-like hallmarks, although the extent of its contribution is varied in a cell type-specific manner. These findings could help our comprehensive understanding of tumor stemness, and also improve the development of eradicative therapies against human malignancies.

## Background

The tumors contain a sub-population of specific cells which are primarily involved in tumor formation and maintenance [1]. Those cells are referred as tumor stem-like cells (TSLCs), and CD133 (also known as prominin-1 or AC133) has been considered as an important marker to enrich the stem-like population in tumors of various tissues, including those of the brain [2], prostate [3], pancreas [4], liver [5], colon [6,7], and skin for melanoma [8]. CD133^+ ^tumor cells possess the ability to self-renew without limit and to generate the majority of differentiated progenies [9]. They are also more resistant to chemo- and radiotherapy when compared to CD133^- ^tumor cells, resulting in tumor progression and recurrence, and thus are considered as a potential therapeutic target to eradicate tumors [10-12]. However, the molecular mechanisms underlying this tumor stemness are still under investigation.

CD133 is a cellular surface glycoprotein containing five transmembrane regions and two glycosylated extracellular loops and has a molecular weight of 97-120 kDa. It was identified as a specific antigen for human hematopoietic stem cells [13], and is currently used for the isolation of stem-like cells from numerous tissues [13-20]. Although little is known about the biological function of CD133, recent studies have shown that prominin-1 null mice were born and aged normally [21], but resulted in progressive degeneration of mature photoreceptors with complete loss of vision after postnatal day 15 [22]. Transcription of *CD133 *gene is known to be controlled by alternative five promoters (P1, P2, P3, P4, and P5), and exon 1 produces different spliced 5'-UTRs, which are expressed in a tissue-specific manner [23]. We previously showed that the methylation status of CpG sites residing in P1 and P2 regions is inversely correlated with the expression levels of *CD133 *mRNA in human glioma tissues [24]; however, any molecules involved in the transcriptional regulation of *CD133 *gene are still unknown.

The E26 transformation-specific (ETS) family consists of over 35 *Ets *genes that can be structurally categorized into 11 subfamilies in humans [25]. Individual Ets genes share a highly conserved DNA-binding domain composed of about 85 amino acid residues referred to as the ETS domain, which recognizes purine-rich GGA(A/T) (ETS binding site; EBS). Several Ets factors have been shown to be nuclear targets for the activation of the Ras/Raf/MEK/ERK signaling pathway and are involved in various biological processes, including cell proliferation, apoptosis, differentiation, hematopoiesis, tissue remodeling, angiogenesis, metastasis, and oncogenic transformation [25]. The altered expression levels of Ets or chromosomal amplification, deletion, and translocation are known to cause human leukemia or specific types of solid tumors [26].

To investigate the molecular mechanism underlying stem-like features in human tumors, we characterized a promoter region of human *CD133 *gene by using human carcinoma and sarcoma cell lines. We found that the ERK pathway is involved in the expression of CD133, and its inhibition was also shown to decrease the frequency of side population (SP), another hallmark of stem-like cells. Finally, it was revealed that Ras-mediated transformed astrocytes have an ability to form greater colonies in the neural stem cell culture condition, together with the increased *CD133 *mRNA expression. Thus, our finding could provide important insights into the molecular basis of tumor stemness.

## Methods

### Cell culture, reagents, and transfection

The human colon carcinoma cell line Caco-2 and synovial sarcoma cell line Fuji were cultured in DMEM supplemented with 20% and 10% fetal bovine serum (FBS), respectively. The immortalized normal human astrocytes NHA/TS and Ras-transformed NHA/TSR cells were previously established [27]. Cells were treated with the MEK inhibitor U0126 (Cell Signaling Technologies, Beverly, MA, USA) at 20 μM for 72 hours with renewal of media every 24 hours. Fugene HD transfection reagent (Roche, Indianapolis, USA) was used for transfection. For primary neurosphere formation, NHA/TS and NHA/TSR cells (1000 cells/3.5 cm dish) were cultured in neural stem cell (NSC) medium (DMEM/F12 containing 20 ng/ml EGF, 20 ng/ml bFGF, 1× B27, and 1% penicillin and streptomycin) for 2 weeks as described previously with minor modification [28]. After 2 weeks of culture, TSR spheres were collected, enzymatically dissociated by Trypsin-EDTA and reseeded at a density of 1000 cells/3.5 cm dish) for secondary sphere formation. For clonal culture, single NHA/TS or NHA/TSR cell was transferred into 48-well plates containing NSC medium using cell sorter. After 2 weeks, each well was manually screened for a colony using phase contrast microscopy. Thereafter, each individual primary TSR sphere was dissociated and reseeded (1000 cells/3.5cm dish) again for secondary sphere formation. For reactivation of CD133 expression, cells were treated with 5 μM 5-Aza-2-deoxycitidine (5-Aza-dC; Sigma, St. Louis, MO, USA) for the initial 48 hours, and then in combination with 500nM Trichostatine A (TSA; Sigma) for an additional 24 hours.

### Plasmids

pCR3.1-Uni-CD133 expression plasmid was kindly provided by Dr. Denis Corbeil [29]. pGL3 enh-P1, P2, and P3 were previously established [24]. To generate pGL3enh-P4 and P5 reporter gene constructs, P4 and P5 regions of human *CD133 *gene were amplified by PCR using genomic DNA isolated from human normal peripheral blood monocytes. Oligonucleotide primers were designed on the basis of DNA sequences {GenBank: AY438641 for P4 and GenBank: AY438640 for P5}. The PCR products were gel-purified, cloned into pCR-Blunt II-TOPO vector (Invitrogen, Carlsbad, CA, USA) verifying sequences, and subcloned into the pGL3 enhancer luciferase reporter plasmid (pGL3enh) (Promega, Madison, WI, USA). The reporter plasmids driven by P5 or its deleted forms (pGL3enh-P5-1068, -768, -368, -98, -25) and mutant plasmids of P5 for ETS binding core sequence (GGAA) such as pGL3enh-P5-98mEBS#1, pGL3enh-P5-98mEBS #2, and pGL3enh-P5-98mEBS #1#2, were constructed by PCR-based methods. The cDNA encoding the dominant negative forms of Ets2 (corresponding to amino acid residues 332-469) and Elk1 (corresponding to amino acid residues 1-168), which lack the transcriptional activation domain, were amplified from human brain cDNA and inserted into XhoI and NotI sites of the pCXN2-Flag expression plasmid [30]. For retrovirus-mediated transfer of *Ets2 *and *CD133 *genes into NHA/TS cells, the each cDNA amplified from human brain cDNA and pCR3.1-Uni-CD133 vector was subcloned into pCX4pur retroviral vector kindly provided by Dr. Tsuyoshi Akagi [31].

### RNA extraction and semiquantitative RT-PCR

RNA was isolated with TRI Reagent (Sigma) according to the manufacturer's instructions. After oligo(dT)-primed reverse transcription of 4 μg of total RNA was conducted, the resulting single-stranded cDNA was amplified by PCR using KOD-Plus DNA polymerase (TOYOBO, Osaka, Japan). The specific primers are listed in Additional file 1, Figure S1.

### Immunoblotting

Immunoblotting was performed as described previously [32]. Briefly, cells were lysed and protein concentration of lysates was determined by Protein Assay reagent (Bio-Rad, Richmond, CA, USA). Thirty μg of proteins were separated by SDS-PAGE and transferred to a polyvinylidene difluoride filter, which was subsequently incubated with Tris-HCl-based buffer (pH 7.4) containing 5% dried nonfat milk for 1 hour at room temperature (RT). Filters were probed with mouse monoclonal antibodies to CD133 (1: 200; Miltenyi Biotec, Auburn, CA, USA), Flag M2 (1: 1000; Sigma), and Actin (1: 2500; Chemicon, Temecula, CA, USA), or rabbit polyclonal antibodies to ERK1 (1: 200; Santa Cruz Biotechnology, Santa Cruz, CA, USA) and phospho-p44/p42 MAPK (1: 1000; Cell Signaling). Bound antibodies were detected with peroxidase-labeled goat antibody to mouse IgG or goat antibody to rabbit IgG and visualized by enhanced chemiluminescence reagents (Amersham Pharmacia Biotech, Freiburg, Germany).

### Luciferase reporter assay

Dual-luciferase reporter assay was performed as described previously [33]. Briefly, cells plated on 24-well plates were transiently transfected with both of *CD133 *promoter-firefly luciferase plasmids and internal control plasmid as pRL-TK (Promega) encoding Renilla luciferase for monitoring transfection efficiency. After 48 hours, luciferase activity was measured, and the ratio of firefly activity normalized with the Renilla activity was calculated for each transfection. In the experiments using Ets dominant negative constructs and U0126, single reporter assay system was conducted to exclude the possibility that pRL-TK control vector could be affected by the change of Ets expression, by normalizing firefly activity to protein concentration of cell lysate for each transfection or treatment.

### Electrophoretic mobility shift assay (EMSA)

Nuclear extracts were prepared as described previously [33]. All synthetic oligonucleotides were annealed and end-labeled by T4 polynucleotide kinase (Promega) with [γ-^32^P]ATP (Amersham). The following 25 bp oligonucleotides (double strand DNA) were used for experiments: EBS1, 5'-ATCAGGCAGGAAGGGTAGAATGCTG-3' (position -62 to -38 of *CD133 *P5 promoter); mEBS1, 5'-ATCAGGCATTAAGGGTAGAATGCTG-3' (position -62 to -38 of *CD133 *P5 promoter with mutated Ets site); EBS2, 5'-TGCTGGGACAGGAAGTAGCTTGGAG-3' (position -42 to -18 of *CD133 *P5 promoter); mEBS2, 5'-TGCTGGGACATTAAGTAGCTTGGAG-3' (position -42 to -18 of *CD133 *P5 promoter with mutated Ets site); Cons. EBS, 5'-GGGCTGCTTGAGGAAGTATAAGAAT-3' (Ets responsive element of the human *erbB2 *promoter [34]); and -25/-1, 5'-GCTTGGAGGTGGGCCTTAGGCTGGT-3' (position -25 to -10 of the *CD133 *P5 promoter without Ets site). Nuclear extracts containing 10 μg of proteins were incubated for 20 minutes at RT with ~ 100,000 cpm of the labeled probe in a final volume of 20 μl of 10 mM Tris-HCl (pH 7.5) buffer containing 50mM NaCl, 1 mM dithiothreitol, 1 mM EDTA, 5% glycerol, and 1 μg poly(dI-dC)poly(dI-dC). For competition experiments, 50-fold molar excess of the cold oligonucleotide was added to the nuclear extracts 10 minutes prior to addition of the labeled probe. The reacted probes were subjected to electrophoresis (250 V for 90 minutes at 4°C) on a non-denaturing, 5% polyacrylamide gel containing 1xTGE (25 mM Tris-HCl, 190 mM glycine, 1 mM EGTA) buffer.

### Magnetic and fluorescence activated cell sorting

For enrichment of CD133-expressing cells, Fuji cells were subjected to immunomagnetic separation using a magnetic activated cell sorting (MACS) CD133 Cell Isolation Kit (Miltenyi Biotec), according to the manufacturer's protocol. Briefly, dissociated cells incubated for 30 minutes at 37°C were labeled with CD133/1 Micro Beads. After washing, labeled cells were loaded onto a column installed in a magnetic field. Trapped cells were collected as CD133high fraction after the column was removed from the magnet. The collected cells were applied to a second column and the purification step was repeated. The flow-through of the MACS column is used as CD133low fraction. To check the expression of CD133, cells were stained with phycoerythrin (PE)-conjugated monoclonal antibodies for human CD133 (CD133/2-PE, Miltenyi Biotec) or isotype control antibody IgG2b-PE (Miltenyi Biotec) (1:10 diluted 4°C for 10 minutes in the dark). After washing, the labeled cells were analyzed by a BD FACS Aria flow cytometer (BD Biosciences, San Jose, CA, USA).

### Side population (SP) analysis

To measure the proportion of SP cells, cells were stained with Hoechst 33342 dye (Molecular Probes, Eugene, OR, USA) as previously described [35]. Briefly, cells (1 × 10^6 ^cells/ml) were resuspended in pre-warmed DMEM with 2% FBS. Hoechst 33342 was added at a final concentration of 5 μg/ml in the presence or absence of 50 μM verapamil (Sigma) and the cells were incubated at 37°C for 90 minutes. After incubation, cells were washed and resuspended in cold DMEM with 2% FBS. Propidium iodide (Molecular Probes) at a final concentration of 1 μg/ml was added to discriminate dead cells. Analyses were performed on a BD FACS Aria SORP flow cytometer (BD Biosciences). The Hoechst dye was excited with the UV laser and its fluorescence was measured with a 405/20 nm band pass filter (Hoechst blue) and a 670 nm long pass filter (Hoechst red). Cells were pre-gated through FSC-W vs. FSC-H, and SSC-W vs. SSC-H dot plot to exclude doublet cells.

### Soft-agar colony formation assay

Colony formation assay was performed as described previously [33]. Briefly, cells (4 × 10^4 ^cells per 6cm-dish) were suspended in 0.36% noble agar, and plated on 0.6% bacto agar. Colonies were stained with dimethylthiazol-diphenyltetrazolium (MTT), and photographed by Fuji LAS-1000 imaging system. Colony numbers were calculated by using Multigauge Colony Count Software.

### *In vivo *tumor formation analysis

Animal procedures were performed according to a protocol approved by the institutional Animal Care and Use Committee at Hokkaido University Graduate School of Medicine. 5 × 10^6 ^cells were mixed with matrigel (BD Biosciences) and subcutaneously injected into female nonobese diabetic/severe combined immunodeficiency (NOD/SCID) mice at 6-8 weeks of age. Tumor formation was assessed at 6 weeks after inoculation.

### Statistical analysis and computer analysis

Comparison between experimental groups was made by Student's t-test. p < 0.05 was considered significant. The TFSEARCH program http://www.cbrc.jp/research/db/TFSEARCHJ.html was used for analysis of possible binding sites of transcription factors.

## Results

### P5 exhibits the highest activity among the five putative promoters of *CD133 *gene

**Figure 1:**
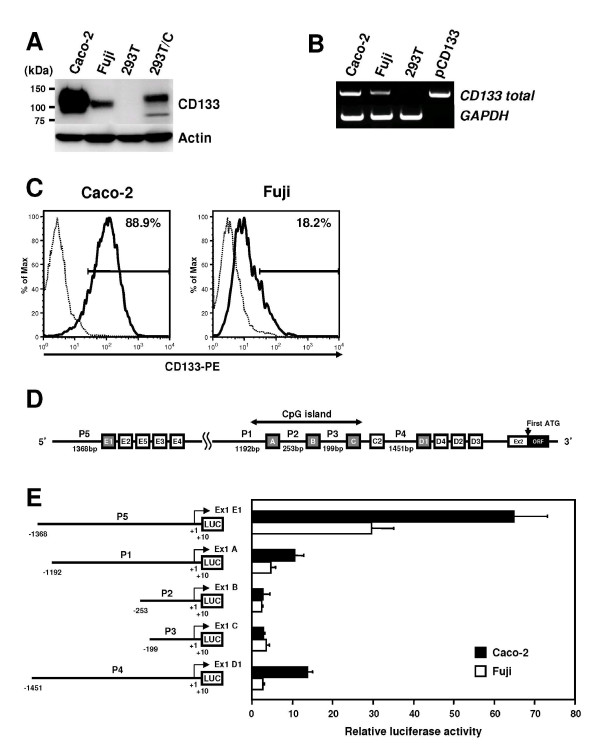
**Expression and promoter activities of *CD133 *gene in human colon carcinoma Caco-2 and synovial sarcoma Fuji cell lines**. **A**, Immunoblot analysis of CD133 protein in Caco-2 and Fuji cell lines. Human kidney cell line 293T and 293T transfected with pCR3.1-Uni-CD133 (293T/C) was used as a negative and a positive control, respectively. Actin is an internal control. **B**, Semiquantitative RT-PCR analysis of *CD133 *mRNA in Caco-2 and Fuji cell lines. cDNA from 293T and 100 ng of pCR3.1-Uni-CD133 plasmid (pCD133) were used as a negative and a positive control, respectively. The number of PCR cycles was at 32 for *CD133*. *GAPDH *is an internal control. **C**, FACS analysis of Caco-2 and Fuji lines with CD133/2-PE antibody. Numbers indicate the percentage of CD133-positive cells in the total population. The bold line represents a staining with CD133-PE antibody, and the dotted line represents the isotype control antibody. **D**, Schematic representation of the position of five *CD133 *promoters (P1 to P5) and exon1s (A to E). Gray boxes represent five first exons starting from the transcriptional start positions. Exons 1C2, 1D4, and 1E5 were identified in our previous study [24]. **E**, Promoter activity of P1, P2, P3, P4, and P5 in Caco-2 and Fuji cell lines. *Left panel*, schematic representation of luciferase reporter plasmids containing five CD133 promoters. +1 indicates the transcription start position of each exon1s. *Right panel*, luciferase activities of Caco-2 and Fuji cells transfected with five indicated reporters. Data are means ± s.d. of values from three independent experiments.

To analyze the transcriptional machinery of *CD133 *gene, we used a human colon carcinoma cell line Caco-2 and a human synovial sarcoma cell line Fuji, because both cell lines express abundant CD133 transcripts and proteins, and also CD133^+ ^Caco-2 cells are reported to be able to generate tumors following transplantation in mice, whereas CD133^- ^cells are not [36]. Our previous studies showed that Caco-2 expresses the transcripts containing all variants of exon 1, and Fuji expresses only exons 1A, 1B, 1C, and 1E (Figure 1D). Immunoblotting and RT-PCR analysis confirmed that the expression levels of CD133 mRNA and protein in Caco-2 cells are higher than those in Fuji cells (Figure 1A, Figure 1B). FACS analysis using anti-CD133 antibody revealed that both cell lines exhibit the cellular heterogeneity for CD133 expression (Figure 1C), as the CD133 positive fraction is 88.9% and 18.2% in Caco2 and Fuji cells, respectively. Luciferase assay demonstrated a remarkable activation of P5 (pGL3enh-P5-1368) and a modest activation of P1 (pGL3enh-P1-1182), and no significant enhancement of P2 (pGL3enh-P2-253) and P3 (pGL3enh-P3-199) was detected in both cells (Figure 1E). A modest activation of P4 (pGL3enh-P4-1451) was observed only in Caco-2 cells, which probably contributes to the expression of exon 1D-containing transcripts.

### Ets binding motifs are required for *CD133 *P5 activity

**Figure 2:**
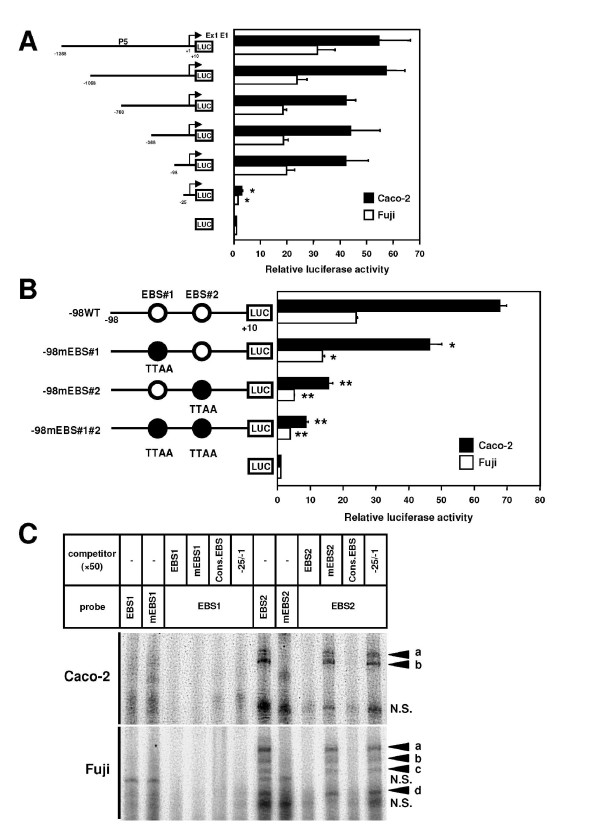
**Identification of Ets binding motifs essential for *CD133 *P5 activity**. **A**, Deletion analysis of the promoter activity of *CD133 *P5. *Left panel*, schematic representation of luciferase reporter constructs containing the deleted sequence of P5 region. +1 indicates the transcription start position of exon1E1. *Right panel*, luciferase activities of Caco-2 and Fuji cells transfected with a series of deletion mutants. Data are means ± s.d. of values from three independent experiments. *P < 0.05. **B**, Effects of mutation of Ets binding sites in the -98/-25 region of the *CD133 *P5 on the transcriptional activity. *Left panel*, schematic representation of P5-98-based luciferase reporter constructs containing point mutation in two Ets binding sites (EBS#1 and EBS#2). Open circles represent wild-type sequence (GGAA) and closed circles represent substituted sequence (TTAA). *Right panel*, luciferase activities of Caco-2 and Fuji cells transfected with the indicated mutant plasmids. *P < 0.05, **P < 0.01. **C**, EMSA analysis of nuclear proteins binding to oligonucleotides containing the Ets binding site of the *CD133 *P5. The arrowheads a to d indicate specific complexes of nuclear proteins with GGAA sequence in EBS#2. N.S.: non-specific binding of proteins.

To determine the minimal region essential for P5 promoter activity, we generated a series of deletion mutants of P5 promoter (pGL3enh-P5-1068, -768, -368, -98, and -25) and found that deletion to -98 holds a similar luciferase activity to the full-length promoter (pGL3enh-P5), but the further deletion to -25 leads to a significant reduction of activity (Figure 2A), suggesting that the region between -98 and -25 contains minimal elements required for *CD133 *transcription through P5 promoter.

The region between -98 to -25 contains two consensus binding motifs of Ets family proteins (G/C)(A/C)GGAAG(G/T) (Additional file 1, Figure S2A) [37]. To determine whether these Ets motifs are necessary for P5 activity, we introduced a substitutional mutation altering two nucleotides of Ets core sequence GGAA to TTAA, into each or both of the Ets binding sites, designated as pGL3enh-P5-98mEBS#1, #2, and #1#2. Introduction of the mutation at EBS#1 moderately decreased P5 promoter activity and the mutation at EBS#2 remarkably decreased the activity (Figure 2B). These results indicated that the two Ets binding motifs are required for the P5 activity, and that in particular, mutation of EBS#2 resulted in the greatest reduction of P5 activity.

### Specific binding of nuclear factors to an Ets motif #2 in *CD133 *P5

Next, to investigate the specific transcription factor which binds to EBS between -98 to -25 of P5, EMSA was performed using nuclear proteins extracted from Caco-2 and Fuji cells. A 25-bp double-stranded oligoDNA containing putative Ets binding sites was synthesized as probes or as unlabeled competitors (Additional file 1, Figure S2B). As shown in Figure 2C, an EBS2 probe, which was designed to span the -42/-18 region containing one proximal Ets motif (EBS#2), showed two major shifted bands following incubation with the nuclear extract from Caco-2 (upper panel, bands a and b), but the EBS1 probe did not. In addition, these signals were eliminated by the use of mutant probes (mEBS2) and by competition using ETS consensus oligonucleotides (Cons. EBS) [34], as well as by 50-fold molar excess amounts of cold wild-type EBS2 probe. -25/-1 oligonucleotides without Ets binding sequence were not able to affect them. These data indicate that the EBS#2, and not EBS#1, is critical for the complex formation on the *CD133 *P5 region. Interestingly, four complexes were obtained using the nuclear extracts from Fuji under the same conditions (lower panel, bands a-d), in which complexes a and b appeared to have similar mobility with those in Caco-2. These results demonstrated that both tumor cell lines express any factors that can bind to the EBS in *CD133 *P5 promoter, although there is a subtle difference in their preferred binding sequence.

### Dominant negative forms of Ets proteins interfere with *CD133 *P5 activity

**Figure 3:**
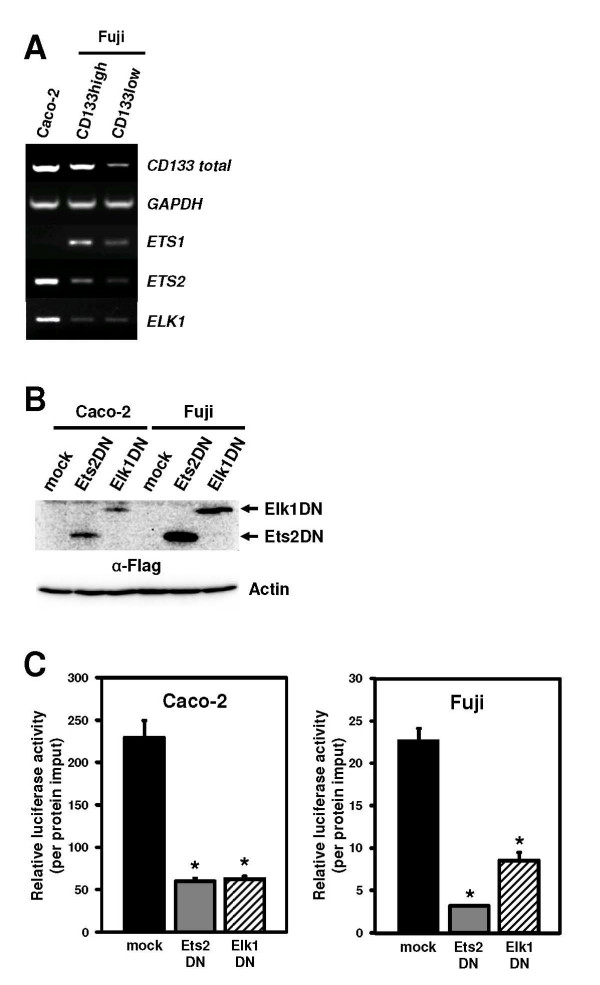
**Dominant negative Ets proteins inhibit *CD133 *P5 activity**. **A**, mRNA expression of *ETS1*, *ETS2*, and *ELK1 *genes in Caco-2 and Fuji cells. Expression pattern of 21 Ets family genes was analyzed by semi-quantitative RT-PCR analysis, in which Fuji cells with a high level of CD133 expression (CD133high) were enriched by MACS. The expression of the other genes except for *ETS1*, *ETS2*, and *ELK1 *are shown in Additional file 1, Figure S3. **B**, Transient overexpression of dominant negative Ets2 (Ets2DN) and Elk1 (Elk1DN) in Caco-2 and Fuji cell lines. Cells were transfected with pCXN2-Flag-Ets2DN and -Elk1DN lacking a transcription activation domain. The expression was evaluated by immunoblot analysis using anti-Flag antibody. **C**, Effects of dominant negative Ets2 and Elk1 on P5 promoter activity. P5 reporter plasmids were co-transfected with dominant negative ETS mutants. Data are means ± s.d. of values from three independent experiments. *P < 0.01 vs. mock-transfected cells.

Ets family proteins share a conserved DNA-binding domain (ETS domain) that recognizes a GGAA sequence. To determine the implication of Ets factors to regulate *CD133 *transcription, the expression levels of 21 human *Ets *genes were examined by semi-quantitative RT-PCR (Figure 3A and Additional file 1, Figure S3), in Caco-2 and Fuji which was separated by the expression amount of CD133 by the MACS system. Ets members were found to be expressed in both tumor lines, but the expression of *ETS1*, *ELK3*, *ER81*, *ERG*, and *FLI1 *genes were not detected in Caco-2 cells. Comparing the CD133high fraction with CD133low in Fuji cells, it was revealed that *ETS1*, *ETS2*, *ELF2*, *ESE1*, *ELF4*, *ERG*, *FLI1*, and *FEV *genes were highly expressed in the CD133-expressing population.

To test whether Ets proteins could specifically mediate P5 transcription, we used a transient transfection approach, employing dominant-negative Ets2 (Ets2DN) and Elk1 (Elk1DN) lacking a transactivation domain. Expression of Ets2DN and Elk1DN resulted in significant inhibition of P5 activity in both cell lines (Figure 3B, Figure 3C). Since the use of dominant negative Ets constructs can broadly interfere with the function of multiple Ets factors [38], studies utilizing dominant negative forms of Ets cannot identify which members of the Ets family are actually important. Therefore, these results indicate that any Ets factors with ETS domain are required for P5 activity. In addition, the partial decrease of P5 activity by EtsDN constructs might reflect the presence of any redundant factors to achieve P5 transcription.

### MEK/ERK pathway is necessary for *CD133 *transcription

**Figure 4:**
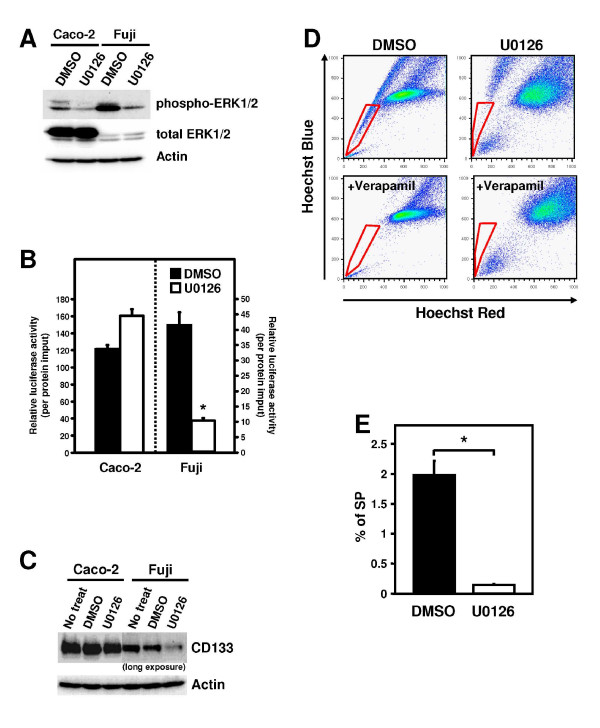
**Inhibition of MEK/ERK pathway attenuates CD133 expression and side population frequency**. **A**, Inhibition of endogenous ERK activation by U0126. Immunoblot analysis was performed with phospho-ERK1/2 and ERK1/2 antibodies. **B**, Effects of U0126 on P5 promoter activity. Cells treated with DMSO or U0126 for 24 hours were transfected with P5 reporter plasmid, and incubated for an additional 48 hours in the presence of U0126. Data are means ± s.d. of values from three independent experiments. *P < 0.05. **C**, Effect of U0126 on *CD133 *protein expression. Caco-2 and Fuji cells were incubated with DMSO or U0126 for 72 hours, and the expression of CD133 protein was analyzed. Actin is an internal control. **D**, Effect of U0126 on the side population frequency in Caco-2 cells. Caco-2 cells were incubated with DMSO or U0126 for 72 hours, stained with Hoechst 33342, and subjected to side population analysis. Hoechst dye was excited with the UV laser and its fluorescence was resolved with a Hoechst blue (y-axis) and Hoechst red (x-axis). Red boxes are defined as SP fractions with low intensity of Hoechst staining. **F**. Bar graph representation of the percentages of side population cells in Caco-2 cells. Data are expressed as the means ± s.d. for three independent experiments. *P < 0.01.

As several Ets factors, including Ets2 and Elk1, have been shown to be activated through phosphorylation by ERK1/2 (also known as p44/42 MAPK), we examined the contribution of the ERK pathway to *CD133 *gene expression. ERK1/2 were constitutively activated without the external stimuli in both tumor cell lines, and this phosphorylation of ERK1/2 was diminished by the treatment of U0126 (Figure 4A), a potent and specific inhibitor of MEK1/2, which binds to MEK1 and MEK2 regardless of its activation state to inhibit ERK1/2 phosphorylation noncompetitively. Furthermore, to investigate the involvement of MEK/ERK signaling in P5 activity, reporter analysis was performed in the presence or absence of U0126. Treatment of Fuji cells with U0126 led to the marked inhibition of P5 activity (Figure 4B). No cell toxicity was observed concerning morphology and growth of both cell lines under the conditions of this experiment (data not shown). Consistently, U0126 also markedly decreased the expression of CD133 protein in Fuji cells (Figure 4C), indicating that the MEK/ERK signaling is implicated in P5-mediated CD133 expression. In contrast, the same concentration of U0126 could not affect P5 activity and expression of CD133 in Caco-2 cells, suggesting that another pathway could regulate Ets-mediated P5 transcription in Caco-2 cells. However, it remains possible that the ERK pathway might simultaneously regulate other promoters (P1 to P4). In fact, ERK inhibition decreased the expression of exon 1B- and 1D- and 1E-containing CD133 mRNA, but increase exon 1C-containing one (Additional file 1, Figure S4). These data indicate cell type-specific regulation of *CD133 *gene expression by the ERK pathway.

### Inhibition of MEK/ERK pathway abolishes side population in Caco-2 cells

To assure the relevance of the ERK pathway to stem-like characteristics, the effect of U0126 on the amount of SP was assessed by flow cytometric analysis. The SP fraction in tumor cells has been known to define the population containing stem-like cells, which highly express ATP-binding cassette (ABC) transporters to efflux both Hoechst dye and chemotherapeutic agents, and to have a high capacity to form tumor xenografts in mice [39]. In our experiments, the side population represented approximately 2% in the Caco-2 cell line (Figure 4D, Figure 4E). Treatment with verapamil, an inhibitor of the ABC transporters, completely ablated this population. In contrast, no distinct SP was visible in the Fuji cell line (data not shown). Treatment of Caco-2 with U0126 dramatically reduced the SP frequency to 0.15% (Figure 4D, Figure 4E). This result emphasized our conclusion that ERK is a key molecule in the signal transduction to maintain stem-like features in tumor cells.

### Ets2 increases *CD133 *mRNA levels in human astrocytes, but cannot confer tumorigenicity

**Figure 5:**
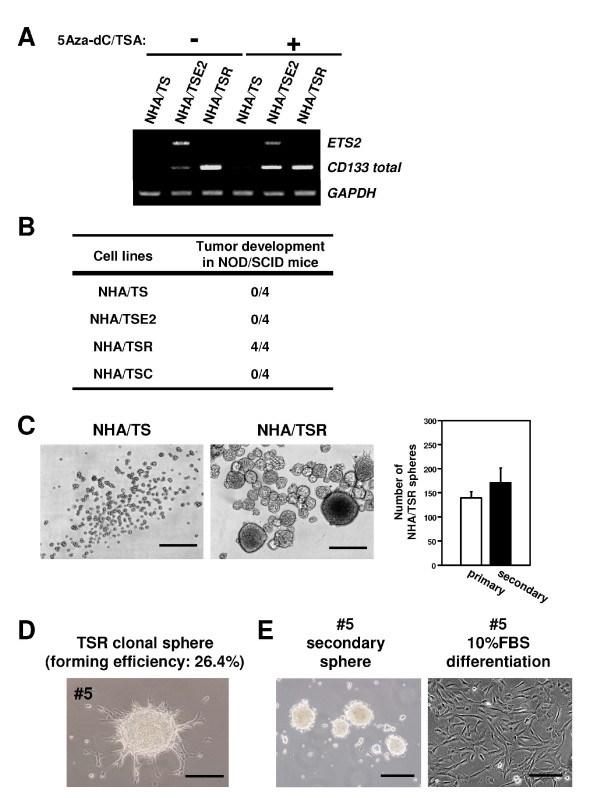
**Ras-transformed astrocytes have an ability to form neurosphere-like colonies with the increased expression of *CD133 *mRNA**. **A**, Expression of *CD133 *mRNA in NHA/TSE2 and NHA/TSR cells. The expression levels of *CD133 *mRNA were examined by semi-quantitative RT-PCR analysis. The number of PCR cycle was 32 for *CD133*. *GAPDH *is an internal control. 5Aza-dC; 5-Aza-2-deoxycitidine. TSA; Trichostatine A. **B**, *In vivo *tumor formation analysis. 5 × 10^6 ^of each cells were subcutaneously injected into NOD/SCID mice. Tumor formation was evaluated after 6 weeks of inoculation. The number of mice developing tumors/numbers of mice injected is shown. **C**, Representative images of NHA/TS and NHA/TSR cells cultured in neural stem cell medium. Bar graph shows mean ± s.d. of the absolute number of primary and secondary TSR spheres, which was measured from three independent experiments. Cell clusters ≥ 50 μm in diameter were counted as spheres. Secondary sphere assay was also performed at the same condition. Scale bar = 200 μm. **D**, Representative images of a sphere derived from single NHA/TSR cell. FACS-purified single NHA/TSR cell was cultured in NSC medium in a 48-well plate for 2 weeks. **E**, Individual TSR-derived clonal sphere was dissociated and reseeded in NSC medium (left) and in 10%FBS-DMEM (right) to confirm the secondary sphere formation activity and differentiation potential, respectively. Scale bar = 200 μm.

To examine whether Ets factor could increase CD133 expression and confer tumorigenicity in normal cells, we established the immortalized human astrocytes overexpressing Ets2 (NHA/TSE2). The increase of *CD133 *mRNA expression was detected in NHA/TSE2 comparing to NHA/TS cells and its effect was strongly enhanced by the treatment with demethylating agent 5-Aza-dC and histone deacetyltransferase inhibitor TSA (Figure 5A). 5Aza-dC/TSA treatment has been shown to open the chromosomal region to increase accessibility for transcription factor complexes to assemble at the promoter and drive gene transcription. However, the elevated protein level of CD133 could not be detected by FACS analysis (Additional file 1, Figure S5). Instead, approximately 0.04% of side population diminished by verapamil was observed in the NHA/TSE2 cells, whereas substantial SP was not visible in NHA/TS cells (Additional file 1, Figure S6). To assess the potential of Ets2 on tumorigenicity, NHA/TSE2 cells were subcutaneously implanted into NOD/SCID mice. All positive control mice implanted with 5 × 10^6 ^NHA/TSR cells, which is a transformed astrocytes [27], developed tumors at 4-6 weeks with 100% incidence (4/4), whereas any of the mice injected with NHA/TS or NHA/TSE2 cells could not grew as tumors at least by 6 weeks (0/4) (Figure 5B). These data indicate that Ets2 is not sufficient to fully transform normal human astrocytes, and thus the acquisition of tumor stem-like characters may be accomplished by synergistic actions of multiple factors.

### Human astrocytes transformed by oncogenic Ras can form neurosphere-like colonies with the increased expression of *CD133 *mRNA

NHA/TSR cells form malignant tumors with features of human anaplastic astrocytoma, the WHO classification Grade III in mice [27] and malignant astrocytoma has been well-known to display a stem cell signature [2]. To confirm whether Ras-mediated transformation in human astrocytes could confer stem-like features in parallel with the acquisition of tumorigenicity, we examined the expression level of CD133 and the activity of neurosphere formation in NHA/TSR cells. In the absence of 5Aza-dC/TSA, the more substantial level of *CD133 *mRNA was detected in NHA/TSR cells comparing to NHA/TSE2 cells (Figure 5A), suggesting that Ras might play an additional role to modulate chromatin structure of *CD133 *promoter region. Actually, CD133 expression is regulated by promoter DNA methylation [24]. However, the protein level of CD133 could not be increased (Additional file 1, Figures S5 and S7), suggesting that Ras activation is also not sufficient to completely restore CD133 expression in human astrocytes. The generation of neurospheres is considered as an important symbol of *in vitro *neural stem cell culture, as well as an exhibition of self-renewal capacity [40]. After culturing in NSC medium containing bFGF and EGF for 2 weeks, NHA/TSR cells generated significantly greater neurosphere-like colonies (diameter is ≥ 50 μm) than NHA/TS cells, and this capacity was maintained to generate secondary neurospheres (Figure 5C). To except the possibility of cellular aggregates, single NHA/TSR cell was also sorted using flow cytometry and deposited into 48-well plates containing NSC medium. NHA/TSR had a forming efficiency of 12.7 ± 1.5 (26.4%) single spheres per 48-well plate, as opposed to zero for NHA/TS (Figure 5D). A single sphere could also maintain the capacity to form secondary spheres, as well as differentiate into adherent cells morphologically identical to original NHA/TSR cells in 10% FBS-containing medium (Figure 5E), indicating that constitutive activation of the Ras pathway can trigger the development of stem-like population with self-renewing ability to retain stemness, and possibly that human astrocytes could dedifferentiate to premature state following to the Ras-mediated transformation and/or its sequential combination with immortalization. Side population size was not increased in NHA/TSR cells (Additional file 1, Figure S6). These results indicate that the expression of CD133 protein and appearance of side population are not necessarily required for other tumor stem-like behaviors, at least for the capacity to form neurospheres *in vitro *and tumors *in vivo*. Finally, NHA/TS cells retrovirally overexpressed *CD133 *gene (NHA/TSC) could form a larger number of colonies comparing to NHA/TS cells in soft-agar colony formation assay (Additional file 1, Figure S7). However, their number and size are much less than those of NHA/TSR even after they were cultured for 3 weeks. In addition, mice subcutaneously implanted with 5 × 10^6 ^NHA/TSC cells could not develop any tumors at least by 6 weeks (Figure 5B), supporting the view that CD133 is just a concomitant marker for tumorigenic process.

## Discussion

Traditionally, therapeutic procedures for human cancer have been performed based on the implicit understanding that the tumor population is homogeneous. However, emerging evidence has suggested that tumors are hierarchically organized and the capacity of tumor propagation depends mainly on a sub-population of stem-like cells. The discovery of stem-like cells in solid tumors convincingly accounts for chemoresistance, and recurrence arose in a number of human cancers. Many studies have been carried out using stem-like population enriched by a stem cell marker CD133, and these have demonstrated an increased resistance of CD133^+ ^stem-like tumor cells to treatment with chemotherapeutic agents compared with CD133^- ^tumor progenies. In addition, the side population has been also used as one of the methods to enrich the stem-like tumor cells, as well as normal stem cells, and is defined by Hoechst dye exclusion property. Although it remains to be clarified whether the expression of CD133 and transporter molecules directly contribute to tumor progression, the regulatory mechanism of stem-related gene expression could help our understanding of tumor stemness and should be investigated further to improve the development of eradicative therapies against human malignancies.

Previously, we and other investigators reported that the expression levels of *CD133 *mRNA are positively correlated with tumor stage and the poor prognosis of patients [24,41-47]. However, it is still controversial whether CD133 is just a concomitant marker for tumorigenic process or whether it directly leads to tumorigenesis. To examine the role of CD133 expression in normal cells, we established NHA/TSC cells and found that overexpression of CD133 is not sufficient to induce tumorigenic transformation *in vivo *(Figure 5B and Additional file 1, Figure S7). Interestingly, a recent study using genetically engineered mice suggested that CD133 is just a concomitant marker of stem-like cells. Tumors had developed throughout the entire intestine when Wnt signaling was selectively activated in CD133^+ ^or Lgr5^+ ^adult small intestinal stem cells [21,48]. In contrast, carcinomas with lower malignancy were found in less than one in five mice when the same system was targeted to non-stem cells [48]. Therefore, it is conceivable that malignant expansion of tumors depends on the latent potential of adult stem cells to proliferate or differentiate, and thus the increase of *CD133 *mRNA in a higher stage of tumors might be a result of unregulated expansion of CD133^+ ^stem-like cells. Considering that NHA/TSR cells possess the high activity to form neurospheres and tumors in mice without entire expression of CD133 protein (Figure 5), these data would support the view that CD133 is just a marker of stem-like cells.

The expression mechanisms of *CD133 *gene have not been examined so far, despite its expression being recognized as an important stem-related biomarker for a number of different cell lineages, probably because CD133-expressing cells are a very rare sub-population for transcriptional analysis. In addition, we observed some culture effects, in that serial passages of primary glioblastoma culture easily diminished CD133 expression (data not shown). The two tumor cell lines used in this study stably preserve a high proportion of CD133^+^-proliferating cells, and they could be useful tools to further investigate the expression machinery for CD133.

In Figure 1D, we have shown that P5 promoter exhibits the highest activity among the five alternative promoters, but it should be noted that P5 does not necessarily predominate the CD133 expression. First, the stability and translational efficiency of *CD133 *mRNA might be varied by 5'-UTR sequences containing exon1s (1A, 1B, 1C, 1D, or 1E). Indeed, the modulation of 5'-UTR involves in the regulated expression of some proteins regulating growth and differentiation of normal stem cells and plays a role in the progression of specific types of cancers, such as leukemia and prostate cancer [49]. Therefore, the role of each exon1 on CD133 expression needs to be determined. Experiments should be limited to the use of the whole locus including all of exon1s for reporter assay.

Second, epigenetic modifications such as DNA methylation and histone modification have been reported to play important roles in the regulation of various genes [50]. However, it is questionable that the physiological status of chromatin complexes is accurately reconstituted in the transient reporter assay system. Indeed, we reported that the expression levels of *CD133 *mRNA were dramatically restored by the treatment of glioma cell lines with the demethylating agent 5-azacytidine and/or histone deacetylase inhibitor valproic acid [24]. Therefore, it might be possible that epigenetic modification is the final determinant of *CD133 *gene expression and stem-like features. Further studies are needed to address the molecular mechanisms to epigenetically maintain the active state of *CD133 *gene, containing demethylases of DNAs and/or acethyltransferases of histones.

The Ras/ERK pathway plays a crucial role in transducing signals from various external stimuli to control cell adhesion, proliferation, migration, and survival. It is well-known to be deregulated in some types of human tumors [51]; Ras mutations are found in 45% of colon carcinomas and 90% of pancreatic cancers; Raf mutations are found in two-thirds of melanomas, where TSLCs have been enriched by sorting for CD133 protein expression. The Ras pathway was also reported to be involved in stem cell regulation. Undifferentiated human embryonic stem cells (hESCs) require a growth factor FGF, which transmits signals mainly by the Ras/ERK pathway [52]. Indeed, ERK is active in undifferentiated hESCs and inhibition of the ERK pathway with U0126 caused extensive cell death and differentiation [53].

It is noteworthy that myeloproliferative disorders could be initiated by K-Ras(G12D) in a highly restricted population enriched for hematopoietic stem cells (HSCs) [54]. The expression of activated M-Ras in HSCs initiated leukemogenic transformation [55]. In addition, sophisticated mouse tumor models triggered by oncogenic Ras have demonstrated the contribution of the Ras/ERK pathway to the acquisition of cancer stem cell properties, in primary human mammary epithelial cells (HMECs) [56]. H-RasV12 also causes the p53-knockout mouse-derived astrocytes to be transformed into brain tumor stem-like cells, in which MEK/ERK pathway is responsible for neurosphere formation [57]. Interestingly, *let-7 *microRNA, known to downregulate Ras, reduces proliferation, sphere formation, and the proportion of undifferentiated cells *in vitro *and tumor formation and metastasis *in vivo *[58]. These reports support that the Ras/ERK pathway plays a central role to acquire and maintain tumor stem-like properties.

Furthermore, we have shown that ERK inhibition diminishes the frequency of the side population, but we could not reveal the detailed mechanisms. RT-PCR analysis revealed that the expression of ABCC1 mRNA was decreased by U0126 treatment, while that of ABCG2 mRNA was increased in Caco-2 cells (data not shown). Although it has not been elucidated which transporters have a dominant role for SP phenotype in Caco-2 cells, the ERK pathway might regulate multiple events at the upstream of the SP phenotype, including gene transcription and protein stabilization. Actually, it has been reported that inhibition of the Ras/ERK pathway also promotes ABCB1 protein degradation to diminish the cellular multidrug resistance in the human colorectal cancer cell lines [59]. SP cells are also known to reproduce NSP cells [35]. Therefore, U0126 might affect the division patterns of SP cells via the decrease of self-renewal and/or the increase of differentiation. In the future, we will clarify these points, and should examine whether the therapeutic approaches to Ras/ERK inhibition could be effective for eradication of stem-like cells in tumor mass.

In this study, we found that the Ras/ERK pathway is implicated in at least one of the stem-like characteristics in all of the examined tumor cell lines: SP size in Caco-2, CD133 expression in Fuji, and sphere and tumor forming activity in NHA/TSR cells. These finding suggest that Ras/ERK could be a common upstream pathway to govern the entirety of downstream characteristics. However, the contribution of Ras/ERK was varied in a cell type-specific manner; Ras/ERK does not contribute to CD133 protein expression in Caco-2 and NHA/TSR, and SP appearance in Fuji and NHS/TSR cells. Crosstalk between ERK and other pathway specific to an individual type of tumors may result in the diversity of stem-like hallmarks.

## Conclusions

This study has characterized the critical cis-acting element in *CD133 *promoter P5 and identified one potential pathway required for *CD133 *gene regulation, side population maintenance, and spheres forming activity in stem cell culture. As CD133 expression is now recognized as one of the most important biomarkers for the enrichment of stem-like tumor cells, the Ets motifs and Ras/ERK pathway we identified in this study could lead to a more comprehensive understanding of the molecular basis of tumor stemness. Further characterization of the five *CD133 *promoters, including protein-protein interactions and epigenetic regulations, must allow us to identify *bona fide *targets that define tumor stemness, and facilitate the development of TSLC-targeted therapies to eradicate human malignancies.

## Competing interests

The authors declare that they have no competing interests.

## Authors' contributions

KT designed the research, performed most of the experiments and data analyses, and drafted the manuscript. TK carried out MACS to separate CD133high and low Fuji cells and helped animal experiments. KS helped soft agar assay with human astrocytes and the editing of manuscript. LW helped cell cultures and plasmids extractions and performed animal experiments. NB helped ChIP assay. HN, TT, and ST contributed to the design of the entire study and the editing of the manuscript. All the authors have read the manuscript and agreed with its content.

## Supplementary Material

Additional file 1 **Additional Figures**. Figures S1-7 as referred to in the main text. Click here for file
