# Supplementary material for: Analysis of an alternative human CD133 promoter reveals the implication of Ras/ERK pathway in tumor stem-like hallmarks
Source: Mol Cancer. 2010 Feb 19;9:39. doi: 10.1186/1476-4598-9-39 (PMC2836276; doi:10.1186/1476-4598-9-39)
Supplement: Additional file 1 — Additional Figures. Figures S1-7 as referred to in the main text. [file 1476-4598-9-39-S1.pdf]

Targets	Primer Sequences (5'→3')	Expected size of product (bp)	PCR annealing temperature (℃)	Number of cycles
CD133	sense: TGGCAACAGCGATCAAGGAGAC<br>antisense: TCGGGGTGGCATGCCTGTCATA	633	60	30-32
GAPDH	sense: CTCATGACCACAGTCCATGC<br>antisense: TTACTCCTTGGAGGCCATGT	489	58	30
ETS1	sense: GCATGCCCAGTGTGTTCCAC<br>antisense: AGGGCAGCAGCAGGAATGAC	483	63	30
ETS2	sense: GCCAGTCGTCCTTGCTGGAT<br>antisense: AGGATGGCGTGCAGTTCCTC	490	63	30
ELK1	sense: GTGAGCGGCCAGAAGTTCGT<br>antisense: CCCAAACCCGGCTCCACATTA	497	63	35
ELK3	sense: ATTCCCTGCAGAACCCACCA<br>antisense: GCCTTTGGGGGAAGAGATGG	427	63	30
ELK4	sense: AACTCAGCCGAGCCCTCAGA<br>antisense: CGGTGGCTTTTTGGAAGGTG	428	63	30
ELF1	sense: CATGGTTGTTGCCCCAGTCA<br>antisense: CCAAGCGCTGACCTTCCACT	464	63	30
ELF2	sense: TCCAACAACAGCGACCTCTCC<br>antisense: AGCTGAAGGCGCACTGACAA	457	63	30
ESE1	sense: GCCCCAGTTCTGGTCGAAGA<br>antisense: GTGGGATCCAGGTCCACGTC	477	63	30
ELF4	sense: TGGACCCAGCGAGAGAAAGG<br>antisense: TGCTGGAGAGACGAGCATGG	465	63	30
ESE2	sense: GGGAGTGGCTCCAGTTCTGC<br>antisense: TTGCCAGGGCTTCCGATTTA	429	63	30
ER81	sense: TCATGGCCTGCCACTGAAAA<br>antisense: TGGTGGGAAGGGGATGTTTG	430	63	30
ER71	sense: TGCACAGCCTGGGACTCTTG<br>antisense: GCCACAGCTGAATGGGACCT	448	63	35
PEA3	sense: CCCCTGGACATTTGCCACTC<br>antisense: AAATGCACCGACCCCTTCCT	426	63	30
ERM	sense: CCCCTTACCAGAGGCGAGGT<br>antisense: AGGAGGTAAGCGGGGCTGTC	403	63	30
TEL	sense: GAGCGCTCAGGATGGAGGAA<br>antisense: GCGGTGCAACAGTTCAATGG	428	63	30
ETV7	sense: CCCACCCAGCACTCTCCAGT<br>antisense: GAGTTTCTGCCCCGGTTCCT	498	63	30
ERG	sense: ATCACCTGGGAAGGCACCAA<br>antisense: TATGGCTGGTGGGGAGCCTA	424	63	30
FLI1	sense: GCGAGTCCAACCCCATGAAC<br>antisense: TGCCCCAAGCTCCTCTTCTG	411	63	30
FEV	sense: AAAGGCAGCGGACAGATCCA<br>antisense: GCGGCCATGAGGTTGAGTTT	425	63	35
GABPA	sense: ACGCGCCAGCTGAATGTGTA<br>antisense: CAGTGCAGCAGCCCATCTTG	416	63	30
EHF	sense: CCGGCAACAACCTCCTTCAC<br>antisense: GAGATCTGAGCCCGGCAGAA	478	63	30

Figure S1. Oligonucleotide primers and PCR conditions used for RT-PCR analysis

A

-98 CAGTGTCTCCCCAGAGAGTGGCTGTTCCCAGTGTCAA -62

-61 TCAGGCA**GGAA**GGGTAGAATGCTGGGACA**GGAA**GTAG -25

EBS#1 EBS#2

+1 Exon1E1

-24 CTTGGAGGTGGGCCTTAGGCTGGTAGAAGTTGCT +10

B

**EBS1:**	−62	ATCAGGCA**GGAA**GGGTAGAATGCTG	−38
**mEBS1:**	−62	ATCAGGCA**TTAA**GGGTAGAATGCTG	−38
**EBS2:**	−42	TGCTGGGACA**GGAA**GTAGCTTGGAG	−18
**mEBS2:**	−42	TGCTGGGACA**TTAA**GTAGCTTGGAG	−18
**Cons. EBS:**		GGGCTGCTTGA**GGAA**GTATAAGAAT	
**−25/−1:**	−25	GCTTGGAGGTGGGCCTTAGGCTGGT	−1

Figure S2. Nucleotide sequence of CD133 P5 -98/+10 region and EMSA probes

A. Nucleotide sequence between -98 to +10 of CD133 P5 region. Two Ets binding sites (EBS#1 and EBS#2; GGAA) are showed in bold letters. The oligonucleotides corresponding to positions -62 to -38 and -42 to -18 were used in the EMSA experiment as probes EBS1 (underlined) and EBS2 (dot-underlined). **B**. Sequence of oligonucleotides used in EMSA experiment. Ets core sequence GGAA is substituted by TTAA in mEBS1 and mEBS2. Cons. EBS is a consensus Ets binding sequence derived from the human erbB2 promoter [34]. Nucleotide sequence between -25 to -1 of CD133 P5 region was used as a negative control without GGAA (-25/-1).

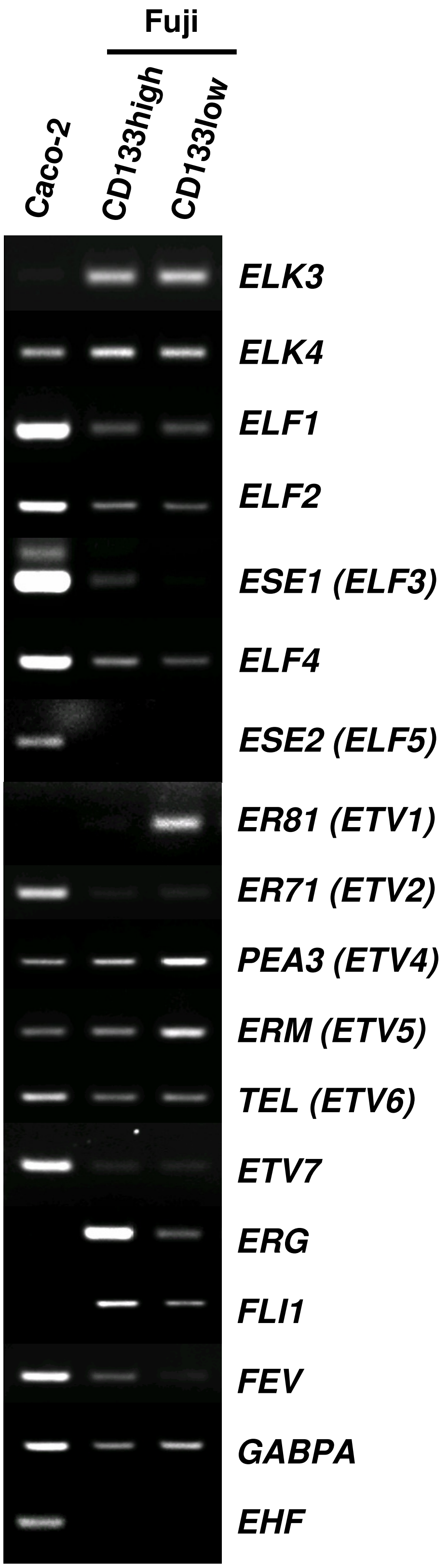

Figure S3. mRNA expression of ETS family genes in Caco-2 and Fuji cells
Primer sequences and PCR conditions are shown in Additional file 1, Figure S1.
Fuji cells with high level of CD133 (CD133high) were enriched by MACS.

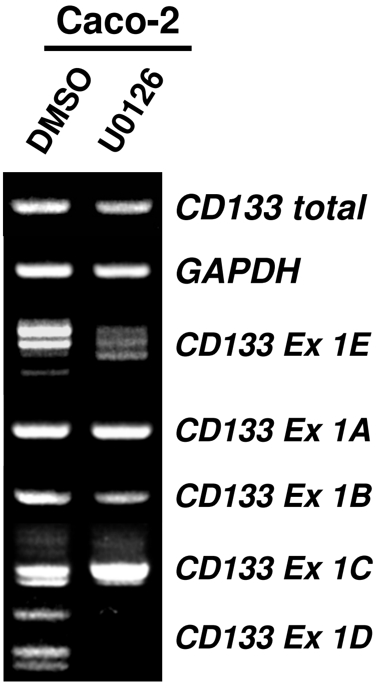

Figure S4. Effect of U0126 on the expression of CD133 mRNA in Caco-2 cells
Cells were incubated with DMSO or U0126 for 72 hours, and the expression of *CD133* transcripts containing exons 1A, 1B, 1C, 1D and 1E were analyzed. PCR amplification was performed using five forward primers located in each of the exon 1s and reverse primer located in the common exon 2 of *CD133* gene. The number of PCR cycle was 25 for total *CD133*, 30 for *exon 1A* and *1B*, and 40 for *exon 1C*, *1D*, and *1E*, respectively. *GAPDH* is an internal control.

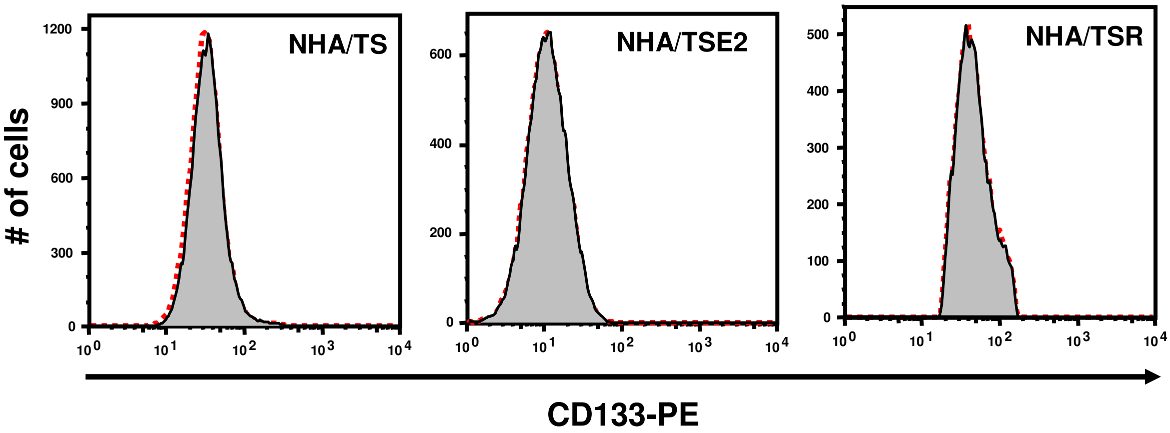

Figure S5. CD133 protein expression in NHA/TSE2 and NHA/TSR cells
FACS analysis was performed using CD133/2-PE antibody. The gray region represents a staining with CD133-PE antibody, and the dotted red line represents the isotype control antibody IgG2b-PE. CD133-positive cells were not detected.

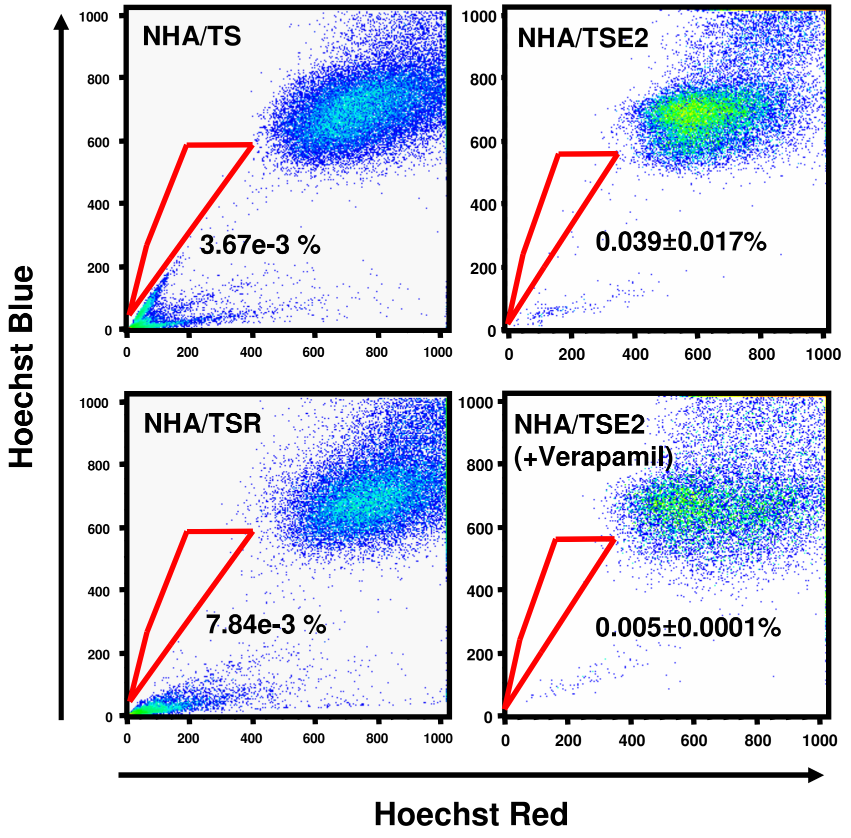

Figure S6. SP analysis of NHA/TSE2 and NHA/TSR cells
Cells were stained with Hoechst 33342, and its fluorescence was resolved with a Hoechst blue (y-axis) and Hoechst red (x-axis). Red boxes are defined as SP fractions with low intensity of Hoechst staining. SP cells were visible in only NHA/TSE2 cells.

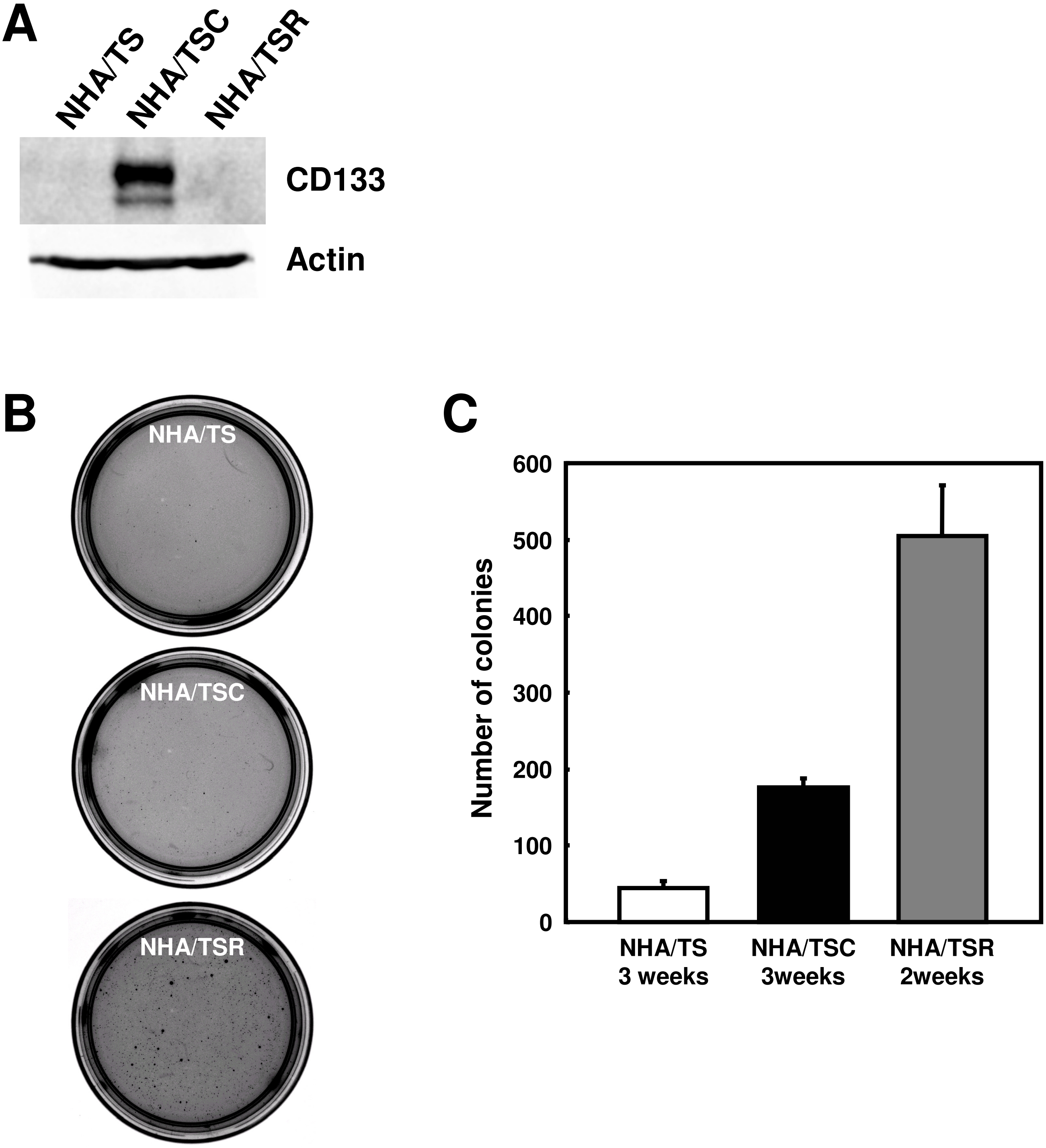

Figure S7. Effect of CD133 expression on soft-agar colony formation activity of NHA/TS

NHA/TS cells infected with retroviral vectors encoding CD133 (NHA/TSC) were established (**A**), and subjected to soft-agar colony formation assay (**B** and **C**). Note that NHA/TSR cells were cultured for only 2 weeks, whereas NHA/TS and NHA/TSC cells were cultured for 3 weeks.
